# Families with Mentally Ill Parents and Their Partners: Overlaps in Psychiatric Symptoms and Symptom Coping

**DOI:** 10.3390/ijerph20075240

**Published:** 2023-03-23

**Authors:** Silke Wiegand-Grefe, Hannah Warkentin, Bonnie Adema, Anne Daubmann, Reinhold Kilian, Sibylle M. Winter, Martin Lambert, Karl Wegscheider, Mareike Busmann

**Affiliations:** 1 Department of Child and Adolescent Psychiatry, University Medical Center Hamburg-Eppendorf, 20251 Hamburg, Germany; 2 Institute for Medical Biometry and Epidemiology, University Medical Center Hamburg-Eppendorf, 20246 Hamburg, Germany; 3 Department of Psychiatry and Psychotherapy II, Ulm University, 89081 Ulm, Germany; 4 Department of Child and Adolescent Psychiatry, Psychosomatic Medicine and Psychotherapy, Charité University Medicine Berlin, 13353 Berlin, Germany; 5 Department of Psychiatry and Psychotherapy, University Medical Center Hamburg-Eppendorf, 20246 Hamburg, Germany

**Keywords:** families, mentally ill parents, mental illness, relationship, psychiatric symptoms, symptom coping

## Abstract

Partners in families with a mentally ill parent often experience psychiatric symptoms themselves. Recent studies indicate that there might be overlaps in disorder-specific symptom areas between partners and spouses. This study aimed at examining associations in psychiatric symptoms and symptom coping in partners in families with a mentally ill parent, e.g., having a psychiatric diagnosis according to the International Classification of Diseases (ICD-10). Furthermore, a moderation of the psychiatric symptoms of the parent with a mental illness on the association in symptom coping was assumed. Families with at least one parent with a mental illness were recruited into the longitudinal “Children of Mentally Ill Parents” (CHIMPS) trial at seven clinical centers in Germany and Switzerland. In total, 139 families were included in the current study. Psychiatric symptoms were assessed using the Brief Symptom Inventory (BSI), Clinical Global Impression scale (CGI), Global Assessment of Functioning (GAF), and Patient Health Questionnaire (PHQ), while symptom coping strategies were measured using the Freiburger Fragebogen zur Krankheitsverarbeitung (FKV). Regression analyses have indicated an association in psychiatric symptoms between mentally ill parents and their partners concerning psychosocial functioning, somatic, and stress-related symptoms. Additionally, one symptom coping strategy of the partners was predicted by the same strategy of the parent with a mental illness. The results emphasize the importance of screening and providing support to parents burdened by the mental disorder of their partners, especially regarding the children in these partnerships.

## 1. Introduction

In Germany, 41.6 million individuals live in a couple relationship [1]. At the same time, nearly one in three adults suffer from a mental disorder [2]. According to these figures, there are several million people in Germany who live with a partner experiencing psychiatric symptoms. Due to the fact that an illness does not only affect the patient themself but can also have an impact on those surrounding and providing care, partners, as well as children in the families of parents with a mental illness, may represent a vulnerable risk group.

A broad range of studies show that the psychiatric symptoms and mental disorders of a person may pose a risk for the mental health of their romantic partner. The partners and spouses of persons with a mental illness often experience psychiatric symptoms them-selves, show significantly more psychological distress and less psychic wellbeing than the general population as they often report emotional detachment and exhaustion, a loss of self, and symptoms of anxiety as well as depression [3,4,5]. Furthermore, a loss of quality of life and social integration in the partners of persons with a mental illness has been reported [6]. Thomsen et al. (2013) conducted a long-term register-based study of all Danish people between 18 and 70 years of age [7]. The results showed that individuals cohabiting with a psychiatric patient had a higher ratio of falling ill with a psychiatric disorder themselves during a 13-year period compared to persons cohabiting with persons who were not mentally ill. In that survey, the men’s ratio to become ill secondarily was slightly higher than that of women. Patients with schizophrenia and men with bipolar disorder had the highest ratio of living together with a person diagnosed similarly. In addition to psychiatric symptoms, impairments in physical health have been reported in spouses caring for their mentally ill partner [8].

There are a range of hypotheses which attempt to explain the increased risk of developing psychiatric symptoms in the partners of persons with a mental illness. Wittenberg et al. (2013) ascribed the results of their qualitative interview survey whereupon one’s family member’s illness as being associated with negative psychological and somatic health outcomes mostly to so-called “spillover effects” [5]. Based on the consideration that the behavior and wellbeing of a person effects the behavior and wellbeing of surrounding persons, close contact with a mentally ill person can result in an impairment of emotional health. Another explanation for psychiatric symptoms in the spouses of persons with a mental illness is “assortative mating”, which describes a developmental phenomenon stating that species tend to seek out a partner whose features match their own [9,10]. According to this theory, it can be assumed that an individual seeks consistency in close human relations and chooses a partner whose characteristics are similar to their own as far as their mental state as well as in their manner of pursuing and satisfying their needs. Thus, spouses often show more similarity in specific psychological traits or disorders than they would be under random mating [9]. A study investigating members of a total of 4.015 families regarding overlaps in borderline personality disorder components showed significant resemblance in spouses, which was best attributed to phenotypic assortative mating [11]. In a meta-analysis of six studies on assortative mating in affective disorders, the occurrence of a phenomenon has been detected in couples for depression and bipolar disorder, with the effect being even more significant in the case of bipolar symptoms [12].

When attempting to understand the development of psychiatric symptoms in the spouses of persons with a mental illness, it is also important to attend to protective and risk factors. While, for instance, a higher relationship quality and marital satisfaction can be a protective factor against caregiver burden and psychiatric symptoms, marital distress tends to enhance symptoms such as depressive syndromes and vice versa [13,14]. Additionally, the length of the relationship and the chronicity of a disorder can be important, as a prolonged illness can lead to an exacerbation of the symptoms in the person affected and the burden of caregivers [15].

Another crucial factor, when it comes to the development of mental illness, is the individuals’ disease management and coping strategies, meaning the manner in which the stressor, for example, the mental symptoms, is managed [16]. Those strategies can be both exacerbating and mitigating: while approach and active forms of coping are associated with fewer psychiatric symptoms, avoidant forms may lead to an increased risk of illness [17]. The severity of the illness is significantly linked to the coping strategies used, with the more severely ill coping more dysfunctionally [18]. In light of the similarities in psychological traits and symptoms reviewed above, it seems possible that partners show similar behavior patterns for coping with such symptoms. In terms of interdependence in couple relationships, it is assumed that behavior patterns can be transmitted from one partner to the other, especially in longer-term relationships [19]. Therefore, one can suppose that there are also overlaps in the manner of handling the symptoms between partners. This manner in which two persons interact in terms of coping is called dyadic coping [20].

Based on the previous research reviewed above, two research questions have been examined to analyze the associations of (1) psychiatric symptoms and (2) symptom coping in couples, including one mentally ill parent and their partners. 

## 2. Materials and Methods

### 2.1. Sample

In this cross-sectional study, we used baseline data from the project “Children of Mentally Ill Parents” (CHIMPS). The CHIMPS project is a longitudinal randomized controlled trial conducted by the University Medical Center Hamburg-Eppendorf [21]. It was developed in seven clinical centers in Germany and in Switzerland: Hamburg, Leipzig, Gütersloh-Paderborn, Ulm-Günzburg, Wiesbaden-Rheingau, Berlin, and Winterthur. The CHIMPS project has been approved by the Ethics Committee of the Medical Association in Hamburg, Germany, and is registered on ClinicalTrials. 

The total sample of the CHIMPS project recruited in all participating centers consisted of *N* = 216 families. In this study, *n* = 2 families were excluded based on completely missing data on all measurement time points, and *n* = 2 families were excluded from the analyses because of completely missing data at baseline assessment. Furthermore, *n* = 73 families were not included in the analysis due to the mentally ill parent’s participation in the study. The total sample of our analysis consisted of *n* = 139 couples with a mentally ill parent and their partner. We have used the data from the baseline assessment concerning psychiatric symptoms and symptom coping of both mentally ill parents and their partners. 

Inclusion criteria were a family with at least one parent, who was diagnosed with a mental illness according to the International Classification of Diseases (ICD-10) [22] and at least one child between the age of three and nineteen years of age. Furthermore, consent to participate in the study and a sufficient knowledge of the German language were required. Exclusion criteria were severe psychiatric disorders and impairments with acute symptoms such as suicidal tendencies, massive self-injurious behavior, and acute psychotic symptoms, making a stationary treatment inevitable and making an ambulatory intervention appear contraindicated (these patients were placed in stationary treatment).

### 2.2. Measurements

#### 2.2.1. Brief Symptom Inventory (BSI)

The psychiatric symptoms of both partners have been assessed by the self-rating instrument Brief Symptom Inventory (BSI) [23]. The BSI measures the subjectively perceived impairment caused by psychological and physical symptoms over a time period of seven days. The clinically relevant symptoms are identified on a five-point scale from “not at all” to “extremely” over 53 items in nine subscales. An overall psychiatric symptom score is summarized in the Global Severity Index (GSI) which was used in the present study. According to the authors, the internal consistency of the scales amounts to a Cronbach’s *α* = 0.39 to 0.89, depending on the sample, and *α* = 0.92 to 0.96 for the GSI. The retest-reliabilities (after one week) amount to *r* = 0.68 to 0.91 [24]. Further, the BSI can be looked upon as a valid instrument [23]. Regarding the data of the sample used in this survey, the internal consistency of the BSI GSI amounts to a Cronbach’s *α* = 0.97.

#### 2.2.2. Clinical Global Impression Scale (CGI)

The Clinical Global Impression scale (CGI) is an instrument that allows a diagnostician to rate the severity of psychiatric symptoms on a seven-point scale ranging from “normal, not at all ill” to “among the most extremely ill patients” [25]. Regarding the specific item used in the survey, the retest reliability coefficient ranges from *r* = 0.20 to 0.81 [26]. Moreover, validity can be confirmed for the CGI [27].

#### 2.2.3. Global Assessment of Functioning (GAF) 

Similarly, the Global Assessment of Functioning (GAF) was also collected by external raters and was used to assess the psychosocial functioning of an individual depending on the impairment caused by the psychiatric symptoms the person concerned experiences on a daily basis [28]. The impairment by the symptoms is measured on a scale of 1 to 100, with 1 referring to “severely impaired” and 100 implying “extremely high functioning”. In an investigation, the reliability and validity of the GAF have been reported [29].

#### 2.2.4. Patient Health Questionnaire (PHQ)

The Patient Health Questionnaire (PHQ) is a self-rating instrument that uses 78 items to assess the severity of mental disorders and syndromes [30]. Next to a categorial detection of mental disorders, the PHQ allows the evaluation of three scales: Somatic (SOM), depressive (DEP), and stress-related (STRESS) symptoms. Those scales were used in the current study. Somatic and stress-related symptoms are measured on a three-point-scale ranging from “not impaired” to “highly impaired”, the frequency of problems with depressive symptoms are assessed on a four-point scale from “not at all” to “almost every day”. Internal consistency of the scales is ranging from Cronbach’s *α* = 0.79 to 0.88 [31]. Moreover, Löwe et al. (2002) reported the PHQ to be a well evaluated, valid instrument [30]. In this study, the internal consistency of the three scales amounts to a Cronbach’s *α* = 0.47 to 0.84. 

#### 2.2.5. Freiburger Fragebogen zur Krankheitsverarbeitung (FKV)

In order to assess the manner in which the partners cope with the psychiatric symptoms the short version of the Freiburger Fragebogen zur Krankheitsverarbeitung (FKV-LIS) was used [32]. The FKV-LIS is a self-rating instrument based on coping models by Lazarus and Folkman (1984) [33]. With 35 items the FKV-LIS covers five dimensions of cognitive, emotional, and behavioral coping strategies of both partners on a five-point-scale ranging from “not at all” to “extremely”: Ineffective coping strategies such as depressive processing (DEP), religiosity and search for meaning (REL), trivialization and wishful thinking (TRI) as well as effective strategies such as active problem-oriented coping (ACT) and distraction and self-building (DIS). The author reports an internal consistency of Cronbach’s *α* = 0.64 to 0.94 for the five scales. Besides, internal validity can be assumed for the FKV [32]. Regarding the reliability in the present study, the internal consistency of the scales ranges between Cronbach’s *α* = 0.62 and 0.77 for this sample.

#### 2.2.6. Family Status

The current family status of the partners was collected using an item distinguishing between single, divorced, married, and widowed. For the analyses, the family status was dichotomized to no marriage (single/divorced/widowed) and marriage (married).

### 2.3. Statistical Analyses

The descriptive analysis of the data was carried out by calculating the ratios arithmetic mean and standard error of the mean. To determine differences in the values of symptomatology measured with the BSI, CGI, GAF, and PHQ, as well as in the values of symptom coping measured with FKV between the mentally ill parents and their partners, two-tailed paired sample t-tests were performed. In order to estimate the percentage of clinically relevant values in the BSI GSI score, we oriented ourselves by the cut-off values of the German manual [23]. Moreover, we compared the partners’ BSI GSI score with the data of a German non-clinical norm sample (*n* = 600) using a one-sample t-test to assess a potential general increase in psychiatric symptoms [23].

Hierarchical linear regression analyses were used to conduct predictor analyses. Prior to the testing, the data were checked regarding the assumptions for calculating a multiple linear regression. The normal distribution was tested utilizing the Shapiro–Wilk test. Subsequently, we included the following predictor variables to test the first research question: the psychiatric symptoms of the mentally ill parent measured by the BSI GSI, CGI, GAF, and PHQ. The outcome variables were the psychiatric symptoms of the partner, e.g., the BSI GSI, CGI, GAF, and PHQ (model 1.1.–1.6.). For the second research question, the predictor variable was symptom coping evaluated by the FKV of the mentally ill parent. The outcome variable was the FKV from the perspective of the partner (model 2.1.–2.5.). As control variables, the gender of the mentally ill parent as well as their age and the family status of both partners, distinguishing between married and unmarried couples, were used. For a hierarchical approach, we entered the control variables first in every model. Then, in a second step, we included the main predictors into the models. Since the PHQ, CGI, and GAF were added as a measure in the course of the study, sub-analyses with PHQ, CGI, and GAF were conducted with reduced sample sizes. 

The analyses were performed using IBM SPSS Statistics 25 and SPSS Macro PRO-CESS (v3.5) by Andrew F. Hayes. Statistical significance was determined by 95% confidence intervals.

## 3. Results

### 3.1. Sociodemographic and Clinical Statistics

Out of 139 pairs, 88 mothers (63%) and 51 fathers (37%) with a mental illness participated in the study. Those individuals were aged between 23 and 58 years, with an average age of 41 years (SD = 7.17). The age of their partners, 88 fathers (63%) and 51 mothers (37%), ranged from 23 to 59 years. Here, the average age was also 41 years (SD = 7.14). Regarding the family status, most of the couples were married on baseline assessment (*n* = 101; 73%); the others reported being single or divorced (*n* = 38; 27%). 

The ICD-10 diagnoses of the mentally ill parents are presented in Table 1. In regard to BSI GSI, 45% of parents with mental illness and 10% of their partners scored in the range of clinically relevant psychiatric symptoms. Significant differences could be found in the global values BSI GSI (t (138) = 12.60, *p* < 0.001), CGI (t (87) = 18.35, *p* < 0.001), and GAF (t (94) = −14.88, *p* < 0.001). Compared to the scores of a non-clinical norm population (*n* = 600; Franke & Derogatis, 2000), the group of partners showed a significantly higher score in BSI GSI, indicating an increased incidence of psychiatric symptoms compared to the general population (t (138) = 5.53, *p* < 0.001). Further descriptive results of the values measured with the instruments BSI, CGI, GAF, PHQ, and FKV can be found in Table 2.

### 3.2. Prediction of Partners’ Psychiatric Symptoms (BSI GSI, CGI, GAF, and PHQ)

The regression analyses concerning the psychiatric symptoms are shown in Table 3 and Table 4 (model 1.1.–1.6.). 16% of the partners’ BSI GSI score variance could be significantly explained by the BSI GSI of the mentally ill parent (F = 5.23, *p* < 0.001), but the BSI GSI of the parent with mental illness score did not significantly predict the outcome (ß = 0.14, *p* = 0.096). The partners’ BSI GSI score increased with the mentally ill parents’ age (ß = 0.25, *p* = 0.035). Married partners on average had a significantly lower BSI GSI score (ß = −0.30, *p* < 0.001). Partners’ CGI score variance could not be significantly predicted (F = 1.73, *p* = 0.136). The model predicting the partners’ GAF score did not significantly explain the outcome either (F = 2.22, *p* = 0.059). Mentally ill parents’ GAF score was identified as the only significant predictor of the outcome (ß = 0.26, *p* = 0.012). 21% of the partners’ PHQ-SOM score variance could be significantly explained (F = 3.46, *p* = 0.008). The PHQ-SOM score of the parent with mental illness was identified as a significant predictor (ß = 0.36, *p* = 0.003). Married partners on average had a significant lower PHQ-SOM score (ß = −0.34, *p* = 0.005). The model using PHQ-DEP scores significantly explained 18% of the partners’ PHQ-DEP score variance (F = 2.80, *p* = 0.023). The PHQ-DEP score of the parent with mental illness could not be identified as a significant predictor of the outcome (ß = −0.02, *p* = 0.840). Married partners on average had a significant lower PHQ-DEP score (ß = −0.40, *p* = 0.001). 20% of the partners’ PHQ-STRESS score variance could be significantly explained (F = 3.35, *p* = 0.009). The PHQ-STRESS score of the parent with mental illness was identified as a significant predictor (ß = 0.32, *p* = 0.009). Married partners again had a significant lower outcome score (ß = −0.27, *p* = 0.023).

### 3.3. Prediction of Partners’ Symptom Coping (FKV)

The prediction analyses as to the partners’ symptom coping are shown in Table 5 and Table 6 (model 2.1.–2.5.). The model predicting the partners’ FKV-REL score significantly explained 8% of the variance in the outcome (F = 2.44, *p* = 0.038). The FKV-REL score of the mentally ill parent could be identified as the only significant predictor of the outcome (ß = 0.21, *p* = 0.014). In the other four models, the FKV-DEP, FKV-ACT, FKV-DIS and FKV-TRI scores of the parents with mental illness did not significantly predict the variance in the partners’ FKV scores. Regarding significant predictors among the demographic variables, a higher age of the parents with mental illness (ß = 0.28, *p* = 0.032) and a lower age of the partners (ß = −0.30, *p* = 0.015) were significantly associated with the partners’ FKV-DEP score. Further, male partners on average had a significantly lower FKV-ACT score (ß = −0.21, *p* = 0.036). Married partners on average had a significantly higher FKV-DIS score (ß = 0.18, *p* = 0.038).

## 4. Discussion

This study aimed to analyze the associations between psychiatric symptomatology and the symptom coping of parents with a mental illness and of their partners. The results showed the significant predictions of the partners’ symptomatology by the symptomatology of the parent with a mental illness. There were indications for an association in one coping strategy involving religiosity and search for meaning.

The psychiatric symptomatology of the parent with a mental illness was found to be a significant predictor for their partner’s psychiatric symptomatology concerning psychosocial functioning, somatic symptoms, and stress-related symptoms, although the first model regarding psychosocial functioning did not become significant as a whole. Moreover, the partners of parents with a mental illness showed higher scores in overall psychiatric symptoms than a normal population. Contrary to our assumption, there was no significant association in depressive symptoms, overall psychiatric symptoms, or symptom severity between the partners. Consistent with previous research, our findings show that the partners of parents with a mental illness experience more psychiatric symptoms compared to the general population [3,4]. In addition, there were significant overlaps in certain symptom domains, suggesting that partners are burdened in similar symptom domains as reported for posttraumatic and depressive disorders before [34,35].

Previous research identified the different causes of these increased psychiatric symptoms and overlaps in symptom domains in the partners of parents with a mental illness, of which two were examined in detail in this paper. In 2013, the family members of parents with a mental illness reported negative effects on their emotional health to be mostly caused by spill-over effects due to the close contact to the ill person [5]. These effects occurred especially among the partners of persons with a mental illness who reported the widest range of spillover domains. Another explanation for the present findings is the phenomenon of assortative mating, which implies that there have not only been resemblances in psychiatric symptomatology in the partners prior to the relationship, but that these similarities may have even led to the selection of this partner [10]. This phenomenon has already been found to be causal for overlaps in affective symptoms and personality disorder components between romantic partners [11,12].

Due to the cross-sectional nature of the data used in the present study, it remains unclear whether these predictions arise from spillover effects or assortative mating or if there are more complex factors of etiological importance. Regarding demographic variables, marriage significantly predicted lower scores in partners’ symptomatology concerning overall psychiatric symptoms, somatic symptoms, depressive symptoms, and stress-related symptoms, indicating that being married might be a protective factor against the possible negative consequences of one’s partner’s illness. Although the literature reports the possibility of spillover effects [5], it can be assumed that marriage, and thus possibly stronger attachment compared to being divorced or single, can serve as a resource for the partners of people with a mental illness. This study leaves open the question of what exactly constitutes the protective aspect of marriage, but previous research indicates that married couples might have a higher relationship quality than unmarried couples, which has already been found to be a protective factor when it comes to psychiatric symptoms [14,36]. Additionally, the length of the relationship could be a crucial factor as it is significantly linked to relationship quality and mental health outcomes as well [37,38]. There were also indications that a higher age of the parent with a mental illness and a lower age of the partner could be a risk factor for the partners’ psychiatric symptoms. The chronicity of the disorder could be decisive for this result, as a prolonged illness can lead to an exacerbation of the symptoms in the person affected and the burden of caregivers [15], but in some cases, it could also lead to a mitigation of the effects in surrounding persons potentially due to habituation and adaption, as has been already shown in the children of parents with a mental illness [39].

As for the second research question, there have been significant associations in the symptom coping strategy of religiosity and the search for meaning between the partners. The association between the partners’ coping strategies could be explained by the assumption that family members might share the same religious beliefs. Interestingly, although religiosity as measured by the instrument used in this survey, the FKV, is considered a rather ineffective coping strategy, common religious coping has already been reported to be associated with positive marital outcomes [40,41]. The finding that this question could only be confirmed for one of five coping strategies may be explained by the results of Holubova et al.’s (2019) study, which reported that the severity of an illness is significantly linked to the coping strategies used, meaning that persons experiencing more severe symptoms tend to cope in a more ineffective manner [18]. Although the partners showed a higher overall psychiatric symptom score when compared to the general population, they are significantly healthier than parents with a mental illness according to all the used measurement instruments surveying psychiatric symptomatology in this study. Regarding the use of coping strategies, the two partners differed only in their use of ineffective strategies—depressive processing, religiosity and search for meaning, as well as trivialization and wishful thinking—which indicates that these strategies actually are related to the severity of psychiatric symptoms, as reported in the literature [18]. In light of dyadic coping mentioned before, these results could indicate supportive or delegated dyadic coping, where one partner uses effective coping strategies on behalf of both persons, for example, due to an inability to cope effectively caused by the mental overload of the mentally ill person [20]. This use of different strategies may explain why there are hardly any overlaps in symptom coping strategies in this sample. 

Regarding the demographic variables, being the female partner of a person with a mental illness predicted the use of effective, active, and problem-oriented coping strategies. This result is in accordance with Thomsen’s finding (2013) that men might be more influenced by an existing disorder in their surroundings than women [7]. Moreover, a mentally ill parents’ older age and a partners’ younger age significantly predicted the frequency of using coping strategy depressive processing. This result may also be explained by the chronicity or length of the disorder, which can exacerbate the symptomatology in the affected person [15], but can also mitigate the effects on surrounding individuals [39]. Hence, an older age of the mentally ill parent may refer to a prolonged illness and a younger age of the partner may refer to a shorter disorder duration, which may lead to more impairments by symptoms in either case. As symptom severity is linked to the used coping strategies [18], these increased impairments may lead to a more ineffective manner of handling the symptoms in the form of coping strategy depressive coping. 

Some limitations in the study must be addressed. First, we have not included some variables concerning symptomatology, such as the specific diagnoses or the chronicity of the disorder, although these characteristics are of great importance with regard to an impairment of surrounding persons [7,15]. Furthermore, no specific partnership-related variables were collected. According to the current research, there are variables such as relationship quality as well as the length of a partnership and living together with a mentally ill person which could also have a decisive influence on the development of psychiatric symptoms [13,14,37]. Thus, it is recommended to include unmarried couples who have been in a long-term relationship in this study by expanding the marital status variable. In addition, it would be very interesting to examine gender effects regarding same-sex couples in further studies. Last, the study is bound by the limitations of correlational design due to the cross-sectional character of the data. Hence, it is not possible to ascertain directional relationships but only associations between variables. Further research should thus focus on longitudinal data or methods such as confirmatory models explaining direct and indirect effects on the symptomatology and symptom coping strategies of the partners of persons with a mental illness. Hereby, the causes of potential resemblances could also be identified. Qualitative interviews could also help to better understand the results.

## 5. Conclusions

This study highlights the correlations between the psychiatric symptoms of a partner and their psychosocial, somatic, and stress-related symptoms, as well as their coping mechanisms, such as religiosity and a search for meaning. The study also examines how these coping mechanisms are associated with the symptoms and coping strategies of their mentally ill partners. Moreover, the results have shown a higher incidence of psychiatric symptoms in the partners of parents with a mental illness compared to the general population. These findings corroborate current research suggesting that persons with a mental illness tend to choose a partner with similar characteristics in terms of assortative mating or that a mental disorder of a partner can be a risk factor for the development of one’s own psychopathological mental health issues in the form of spillover effects. 

Therefore, this study highlights the importance of an early screening and providing support to the partners of parents with a mental illness. Clinics should devote more attention to these parents in the form of marital or family interventions, as well as addressing the partners own mental health. In regard to the finding that there have barely been any associations found in terms of symptom coping, clinical practice should further focus on supporting existing resources in the form of effective coping strategies, but also shed light on the potential overburdening of partners due to excessive supportive coping. Children living in such families could benefit especially from this increased focus on the family environment of parents with a mental illness. Interventions supporting the partner who is not (yet) ill could compensate for the burden of these children. Therefore, further research is required in this field to examine how exactly the couple dynamics between the parent with a mental illness and their partner affects their children’s mental health.

## Figures and Tables

**Table 1:** Distribution of ICD-10 diagnoses among mentally ill parents (*n* = 139).

ICD-10 Diagnosis	*n*	%
Mental and behavioral disorders due to the use of psychoactive substances (F10–F19)	2	1.4
Schizophrenia, schizotypal and delusional disorders (F20–F29)	3	2.2
Affective Disorders (F30–F39)	84	60.4
Neurotic, stress-related and somatoform disorders (F40–F49)	14	10.1
Disorders of personality and behavior in adult persons (F60–F69)	35	25.2
Behavioral and emotional disorders with onset usually occurring in childhood and adolescence (F90–F98)	1	0.7
At least one comorbid ICD-10 diagnosis	55	39.6

Note: ICD = International Statistical Classification of Diseases and Related Health Problems.

**Table 2:** Clinical statistics of the sample.

Questionnaire		Parents with Mental Illness	Partners	Differences		
	*N*	M (SD)	M (SD)	df	t	*p*
BSI GSI	139	1.35 (0.68)	0.51 (0.53)	138	12.60	<0.001
CGI	88	5.24 (0.94)	2.61 (1.13)	87	18.35	<0.001
GAF	95	57.68 (11.64)	79.85 (12.70)	94	−14.88	<0.001
PHQ-SOM	72	8.76 (5.21)	4.69 (4.93)	71	5.64	<0.001
PHQ-DEP	72	13.39 (5.96)	5.97 (6.05)	71	7.17	<0.001
PHQ-STRESS	72	11.01 (3.95)	6.11 (3.57)	71	8.95	<0.001
FKV-DEP	139	3.24 (0.75)	2.20 (0.72)	138	11.72	<0.001
FKV-ACT	139	3.21 (0.76)	3.29 (0.75)	138	−0.93	0.353
FKV-DIS	139	2.83 (0.68)	2.86 (0.75)	138	−0.33	0.742
FKV-REL	139	2.61 (0.71)	2.42 (0.82)	138	2.23	0.027
FKV-TRI	139	2.59 (1.05)	1.93 (0.87)	138	5.80	<0.001

Note: BSI GSI = Brief Symptom Inventory Global Severity Index; CGI = Clinical Global Impression Scale; GAF = Global Assessment of Functioning; PHQ-SOM = Patient Health Questionnaire Somatic, PHQ-DEP = Patient Health Questionnaire Depressive; PHQ-STRESS = Patient Health Questionnaire Stress-related; FKV-DEP = Freiburger Fragebogen zur Krankheitsverarbeitung depressive processing; FKV-ACT = Freiburger Fragebogen zur Krankheitsverarbeitung active problem-oriented coping; FKV-DIS = Freiburger Fragebogen zur Krankheitsverarbeitung distraction and self-building; FKV-REL = Freiburger Fragebogen zur Krankheitsverarbeitung religiosity and search for meaning; FKV-TRI = Freiburger Fragebogen zur Krankheitsverarbeitung trivialization and wishful thinking.

**Table 3:** Hierarchical regression analyses predicting partners’ symptomatology using BSI, CGI, and GAF scores.

	Model 1.1 (BSI GSI Partner; *n* = 139)				Model 1.2 (CGI Partner; *n* = 88)				Model 1.3 (GAF Partner; *n* = 95)			
	B	95%-CI		ß	B	95%-CI		ß	B	95%-CI		ß
		LL	UL			LL	UL			LL	UL	
Step 1												
Constant	0.74	0.16	1.31		3.04	1.36	4.73		79.97	62.67	97.27	
Gender mentally ill parent	−0.03	−0.24	0.17	−0.03	0.25	−0.34	0.83	0.11	−3.60	−10.00	2.80	−0.14
Age mentally ill parent	0.02	0.00	0.04	0.27 *	0.03	−0.03	0.07	0.16	−0.19	−0.80	0.41	−0.11
Age partner	−0.02	−0.04	−0.00	−0.25 *	−0.03	−0.08	0.02	−0.18	0.15	−0.43	0.73	0.09
Family status	−0.36	−0.55	−0.17	−0.31 **	−0.53	−1.10	0.04	−0.20	5.03	−1.09	11.15	0.17
Step 2												
Constant	0.56	−0.06	1.17		1.96	−0.15	4.06		61.47	39.34	83.60	
Gender mentally ill parent	−0.06	−0.27	0.14	−0.06	0.27	−0.31	0.86	0.12	−2.16	−8.48	4.16	−0.08
Age mentally ill parent	0.02	0.00	0.04	0.25 *	0.03	−0.02	0.08	0.18	−0.11	−0.70	0.48	−0.06
Age partner	−0.02	−0.03	0.00	−0.22	−0.03	−0.08	0.02	−0.20	0.11	−0.46	0.68	0.06
Family status	−0.35	−0.54	−0.16	−0.30 **	−0.52	−1.08	0.04	−0.20	4.23	−1.74	10.21	0.14
BSI/CGI/GAF mentally ill parent	0.11	−0.02	0.23	0.14	0.22	−0.04	0.47	0.18	0.29	0.06	0.51	0.26 *

Note: BSI GSI = Brief Symptom Inventory Global Severity Index; CGI = Clinical Global Impression Scale; GAF = Global Assessment of Functioning; CI = confidence interval; LL = lower limit; UL = upper limit; * *p* < 0.05, ** *p* < 0.01.

**Table 4:** Hierarchical regression analyses predicting partners’ symptomatology using PHQ scores.

	Model 1.4 (PHQ-SOM Partner, *n* = 72)				Model 1.5 (PHQ-DEP Partner, *n* = 72)				Model 1.6 (PHQ-STRESS Partner, *n* = 72)			
	B	95%-CI		ß	B	95%-CI		ß	B	95%-CI		ß
		LL	UL			LL	UL			LL	UL	
Step 1												
Constant	3.69	−4.20	11.57		9.71	0.46	18.95		7.69	2.03	13.34	
Gender mentally ill parent	−0.34	−3.11	2.44	−0.03	−0.72	−3.98	2.53	−0.06	−0.52	−2.51	1.47	−0.07
Age mentally ill parent	0.08	−0.20	0.36	0.11	0.07	−0.26	0.40	0.08	0.09	−0.11	0.29	0.18
Age partner	0.01	−0.27	0.28	0.01	−0.06	−0.39	0.26	−0.07	−0.09	−0.28	0.11	−0.17
Family status	−3.21	−5.84	−0.59	−0.30 *	−5.29	−8.37	−2.21	−0.40 **	−2.13	−4.01	−0.25	−0.27 *
Step 2												
Constant	2.62	−4.86	10.10		9.98	0.28	19.69		4.85	−0.96	10.66	
Gender mentally ill parent	−1.72	−4.49	1.06	−0.17	−0.70	−3.99	2.59	−0.06	−1.42	−3.44	0.60	−0.20
Age mentally ill parent	0.04	−0.22	0.31	0.06	0.07	−0.27	0.40	0.08	0.09	−0.11	0.28	0.17
Age partner	0.03	−0.24	0.29	0.04	−0.06	−0.39	0.28	−0.06	−0.08	−0.27	0.11	−0.15
Family status	−3.68	−6.18	−1.18	−0.34 **	−5.29	−8.39	−2.19	−0.40 **	−2.10	−3.90	−0.29	−0.27 *
PHQ-SOM/-DEP/-STRESS mentally ill parent	0.34	0.12	0.56	0.36 **	−0.02	−0.26	0.21	−0.02	0.29	0.07	0.50	0.32 **

Note: PHQ-SOM = Patient Health Questionnaire Somatic, PHQ-DEP = Patient Health Questionnaire Depressive; PHQ-STRESS = Patient Health Questionnaire Stress-related; CI = confidence interval; LL = lower limit; UL = upper limit; * *p* < 0.05, ** *p* < 0.01.

**Table 5:** Hierarchical regression analyses predicting partners’ symptom coping strategies using FKV-DEP, FKV-ACT, and FKV-DIS scores.

	Model 2.1 (FKV-DEP Partner; *n* = 139)				Model 2.2 (FKV-ACT Partner; *n* = 139)				Model 2.3 (FKV-DIS Partner; *n* = 139)			
	B	95%-CI		ß	B	95%-CI		ß	B	95%-CI		ß
		LL	UL			LL	UL			LL	UL	
Step 1												
Constant	2.33	1.50	3.17		2.82	1.96	3.68		2.55	1.69	3.42	
Gender mentally ill parent	0.10	−0.20	0.39	0.07	−0.31	−0.61	−0.00	−0.20 *	−0.16	−0.47	0.15	−0.10
Age mentally ill parent	0.03	0.00	0.05	0.27 *	0.01	−0.02	0.03	0.07	0.01	−0.02	0.04	0.10
Age partner	−0.03	−0.05	−0.01	−0.30 *	0.01	−0.02	0.03	0.05	−0.01	−0.03	0.02	−0.05
Family status	−0.16	−0.43	0.12	−0.10	0.19	−0.09	0.47	0.11	0.29	0.01	0.57	0.17 *
Step 2												
Constant	2.29	1.26	3.32		2.61	1.67	3.56		2.35	1.34	3.37	
Gender mentally ill parent	0.10	−0.20	0.40	0.07	−0.33	−0.64	−0.02	−0.21 *	−0.13	−0.45	0.19	−0.08
Age mentally ill parent	0.03	0.00	0.05	0.28 *	0.01	−0.02	0.03	0.04	0.01	−0.02	0.04	0.11
Age partner	−0.03	−0.05	−0.01	−0.30 *	0.01	−0.02	0.03	0.06	−0.01	−0.03	0.02	−0.07
Family status	−0.16	−0.43	0.12	−0.10	0.20	−0.08	0.48	0.12	0.30	0.02	0.59	0.18 *
FKV-DEP/-ACT/-DIS mentally ill parent	0.01	−0.15	0.17	0.01	0.09	−0.08	0.26	0.09	0.07	−0.12	0.26	0.07

Note: FKV-DEP = Freiburger Fragebogen zur Krankheitsverarbeitung depressive processing; FKV-ACT = Freiburger Fragebogen zur Krankheitsverarbeitung active problem-oriented coping; FKV-DIS = Freiburger Fragebogen zur Krankheitsverarbeitung distraction and self-building; CI = confidence interval; LL = lower limit; UL = upper limit; * *p* < 0.05.

**Table 6:** Hierarchical regression analyses predicting partners’ symptom coping strategies using FKV-REL and FKV-TRI scores.

	Model 2.4 (FKV-REL Partner, *n* = 139)				Model 2.5 (FKV-TRI Partner, *n* = 139)			
	B	95%-CI		ß	B	95%-CI		ß
		LL	UL			LL	UL	
Step 1								
Constant	1.98	1.02	2.93		2.35	1.34	3.37	
Gender mentally ill parent	−0.20	−0.54	0.13	−0.12	0.21	−0.14	0.57	0.12
Age mentally ill parent	0.01	−0.02	0.04	0.08	−0.01	−0.04	0.02	−0.05
Age partner	0.00	−0.03	0.03	0.02	−0.01	−0.04	0.02	−0.10
Family status	0.16	−0.15	0.47	0.09	0.23	−0.10	0.56	0.12
Step 2								
Constant	1.49	0.48	2.50		2.29	1.21	3.38	
Gender mentally ill parent	−0.22	−0.55	0.11	−0.13	0.22	−0.14	0.58	0.12
Age mentally ill parent	0.01	−0.02	0.04	0.07	−0.01	−0.04	0.03	−0.04
Age partner	0.00	−0.03	0.03	0.01	−0.01	−0.04	0.02	−0.10
Family status	0.12	−0.19	0.42	0.07	0.23	−0.10	0.56	0.12
FKV-REL/-TRI mentally ill parent	0.24	0.05	0.43	0.21 *	0.02	−0.12	0.16	0.03

Note: FKV-REL = Freiburger Fragebogen zur Krankheitsverarbeitung religiosity and search for meaning; FKV-TRI = Freiburger Fragebogen zur Krankheitsverarbeitung trivialization and wishful thinking; CI = confidence interval; LL = lower limit; UL = upper limit; * *p* < 0.05.

## Data Availability

Data available on request from the authors.
